# Correlated Sensory and Sympathetic Innervation Between the Acupoint BL23 and Kidney in the Rat

**DOI:** 10.3389/fnint.2020.616778

**Published:** 2021-01-11

**Authors:** Zhiyun Zhang, Dongsheng Xu, Jia Wang, Jingjing Cui, Shuang Wu, Ling Zou, Yi Shen, Xianghong Jing, Wanzhu Bai

**Affiliations:** ^1^Key Laboratory of Acupuncture and Neurology of Zhejiang Province, Department of Neurobiology and Acupuncture Research, The Third Clinical Medical College, Zhejiang Chinese Medical University, Hangzhou, China; ^2^Institute of Acupuncture and Moxibustion, China Academy of Chinese Medical Sciences, Beijing, China

**Keywords:** kidney, Shenshu (BL23), double fluorescent neural tracing technique, sensory neuron, postganglionic neuron

## Abstract

**Objective**: To investigate the sensory and sympathetic innervations associated with both acupoint “Shenshu” (BL23) and kidney in the rat for insight into the neuronal correlation between the Back-Shu Point and its corresponding visceral organ.

**Methods**: The BL23 and kidney were selected as the representative acupoint and visceral organ in this study, in which their local nerve fibers were examined by using double fluorescent immunohistochemistry with calcitonin gene-related peptide (CGRP) and tyrosine hydroxylase (TH). Meanwhile, their neuronal correlation in the dorsal root ganglia (DRGs), spinal cord, and sympathetic (paravertebral) chain were investigated using a double fluorescent neural tracing technique with Alexa Fluor 488 and 594 conjugates with cholera toxin subunit B (AF488/594-CTB).

**Results**: The local tissue of acupoint BL23 and the fibrous capsule of kidney distributed abundantly with CGRP- and TH-positive nerve fibers, corresponding to their sensory and sympathetic innervation. On the other hand, the sensory neurons associated with acupoint BL23 and kidney were labeled with AF488/594-CTB and distributed from thoracic (T) 11 to lumbar (L) 3 DRGs and from T10 to L2 DRGs, respectively, in which some of them in T12-T13 DRGs were simultaneously labeled with both AF488/594-CTB. Also, postganglionic neurons associated with both acupoint BL23 and kidney were found in the sympathetic chain at the same spinal segments but separately labeled with AF488-CTB and AF594-CTB.

**Conclusion**: Our study demonstrates the neural characteristics of the acupoint BL23 and kidney in the rat from the perspective of neurochemistry and neural pathways, providing an example for understanding the neuronal correlation between the Back-Shu Points and their corresponding visceral organs. These results suggest that the stimulation of the Back-Shu Points may regulate the activities of the target-organs *via* the periphery sensory and sympathetic pathways.

## Introduction

Back-Shu Points are the specific acupoints on the back, which are named as per their anatomical locations adjacent to visceral organs (in terms of Zang-Fu in Traditional Chinese Medicine; Cao et al., 2017). Although this kind of acupoints is commonly used for regulating the disorders of their corresponding visceral organs following the vicinal principle of acupoint selection, there is a lack of clear understanding of their inherent links (Cheng, 2011; Tu et al., 2019; Dai et al., 2020; Shou et al., 2020). Considering the diagnostic and therapeutic roles of Back-Shu Points playing in visceral diseases, viscerocutaneous reflexes have been paid more attention in this field. However, their neuronal correlation remains unclear (Cabioglu and Arslan, 2008; da Silva, 2010; Liu et al., 2010).

To reveal the inherent links between the Back-Shu Points and their corresponding visceral organs in detail, the acupoint “Shenshu” (BL23) and kidney were selected as the representative targets and examined with neuroanatomical approaches in the present study.

First, a double fluorescent immunohistochemistry with calcitonin gene-related peptide (CGRP) and tyrosine hydroxylase (TH) was employed to observe the sensory and sympathetic innervation in the local tissues of acupoint BL23 and the fibrous capsule of the kidney, respectively (Benarroch, 2011; Chakrabarty et al., 2013; Cui et al., 2015; Wang et al., 2019b). Second, the neural elements associated with acupoint BL23 and kidney were traced by using a double fluorescent neural tracing technique with Alexa Fluor 488 and 594 conjugates of cholera toxin subunit B (AF488/594-CTB) for figuring out the neuronal correlation between the acupoint BL23 and kidney. Through the injection of different neural tracers, sensory, sympathetic and motor neurons associated with BL23 and kidney can be observed. These two kinds of effective tools have been successfully applied in the field of acupuncture research (Xu et al., 2016; Wang et al., 2018b; Cui J. J. et al., 2019). By taking their advantages, we expect not only to determine the innervated characteristics of acupoint BL23 and kidney individually but also to outline the neuronal correlation between them *via* the sensory and sympathetic pathways. From the perspectives of neurochemistry and neural pathways, this research could provide valuable references for understanding the inherent links of Back-Shu Points and their corresponding visceral organs at the cellular level.

## Materials and Methods

### Animals

A total of eight young adult male Sprague–Dawley rats (8–10 weeks, weight 180–210 g) were used in the present study. Animals [license number SCXK(JING) 2017-0005] were provided by the National Institutes for Food and Drug Control. All animals were maintained with free access to water and food under controlled conditions with a 12 h light and dark cycle at a temperature of 24 ± 2°C. This study was approved by the ethics committee of the Institute of Acupuncture and Moxibustion, China Academy of Chinese Medical Sciences (reference number 20160011). All surgical procedures were performed following guidelines for animal experiments by the National Institutes of Health Guide for the Care and Use of Laboratory Animals (National Academy Press, Washington, DC, USA, 1996).

### Surgical Procedures and Tracer Injection

The BL23 and kidney were selected as the representative acupoint and visceral organ in this study. The corresponding site of BL23 on the rat was determined by relative anatomy (Xu et al., 2019). The BL23 locates at the same level as the inferior border of the spinous process of the second lumbar vertebra, 1.5 B-cun lateral to the posterior median line on the human (Huang and Huang, 2007). Under the respiratory anesthesia (1.5% isoflurane), a total of 4 μl 1% AF488-CTB (Invitrogen-Molecular Probes, Eugene, OR, USA) was injected subcutaneously and muscularly into the left side of BL23, while 2 μl 1% AF594-CTB was also injected into the left side of the fibrous capsule of the kidney that was exposed by local surgery. Hamilton micro-syringe was used for the injection. To prevent leakage of the solution, the syringe was left in place for an additional 5 min after injection and then withdrawn slowly.

### Perfusion

Three days after injection, the rats were transcardially perfused with 100 ml of 0.9% saline followed by 300 ml of 4% paraformaldehyde in 0.1 M phosphate-buffered solution (PB, pH = 7.4). The local tissues of BL23 (at length of 5 mm and width of 3 mm) and the fibrous capsule of the kidney on the right side were taken for examining the nerve fibers in the regional distribution, while the spinal cord, dorsal root ganglia (DRGs), and sympathetic (paravertebral) chain on the left side were dissected out and stored in 25% sucrose in 0.1 M PB at 4°C for 2 days.

### Section

Serial transverse sections of the skin tissue of BL23 and longitudinal sections of the sympathetic chain were cut at a thickness of 20 μm on a cryostat (Thermo, Microm International FSE, Germany) and mounted on silane-coated glass slides, while the sections of the spinal cord and DRGs were cut in the transverse and sagittal pattern, respectively on a freezing microtome (Microm International HM 430, Thermo, Germany). Also, the fibrous capsule of the kidney was mounted on silane-coated glass slides in a whole-mount pattern.

### Double Fluorescent Immunohistochemistry

The mounted sections of skin tissue of BL23 and the fibrous capsule of the kidney were simultaneously stained by using double fluorescent immunohistochemistry with CGRP and TH. First, the tissues were incubated in a blocking solution containing 3% normal donkey serum and 0.5% Triton X-100 in 0.1 M PB for 30 min and then transferred to mouse anti-CGRP monoclonal antibody (1:1,000, Abcam, Hong Kong) and rabbit anti-TH antibody (1:1,000, Abcam, Hong Kong) for overnight at 4°C. On the following day, after washing three times with 0.1 M PB, the tissues were exposed to donkey anti-mouse Alexa Fluor 594, donkey anti-rabbit Alexa Fluor 488 secondary antibodies (1:500, Molecular Probes, Eugene, OR, USA), and 4′,6-diamidino-2-phenylindole dihydrochloride (DAPI, 1:40,000; Molecular Probes, Eugene, OR, USA) for 2 h of incubation. After washing, the tissues were coverslipped with 50% glycerin for observation. In contrast, the neuronal labeling with AF488/594-CTB in the sections of the spinal cord, DRGs, and sympathetic chain can be directly observed without further staining.

### Observation and Three-Dimensional Reconstruction

The anatomical structure of tissue sections from the spinal cord was based on Paxinos and Watson (2006). Samples were viewed and analyzed under a fluorescent microscope equipped with a digital camera (DP73, Olympus, Japan) or a laser scanning confocal microscope (FV1200, Olympus, Tokyo, Japan) equipped with a digital camera (DP70, Olympus, Tokyo, Japan). Three-dimensional reconstruction of CGRP- and TH-positive nerve fibers were performed using Imaris 7.7.1 software. Final images were processed with Adobe Photoshop/Illustrator CS5 (Adobe Systems, San Jose, CA, USA). The illustration was drawn with Adobe Illustrator CS5.

### Statistical Analysis

Twenty images captured by fluorescent microscope (10× magnification) were randomly selected to measure the length of nerve fibers in the local tissue of BL23 and the fibrous capsule of the kidney by cellSens dimension software. The density of CGRP- and TH-positive nerve fibers were compared according to the ratio of the length of nerve fibers to the local area. Data was expressed with mean ± standard error of the mean. The statistical analysis was performed by two-tailed Student’s *t*-test using the GraphPad Prism software 7.0.

## Results

### The Distribution of CGRP- and TH-Positive Nerve Fibers

**Figure 1:**
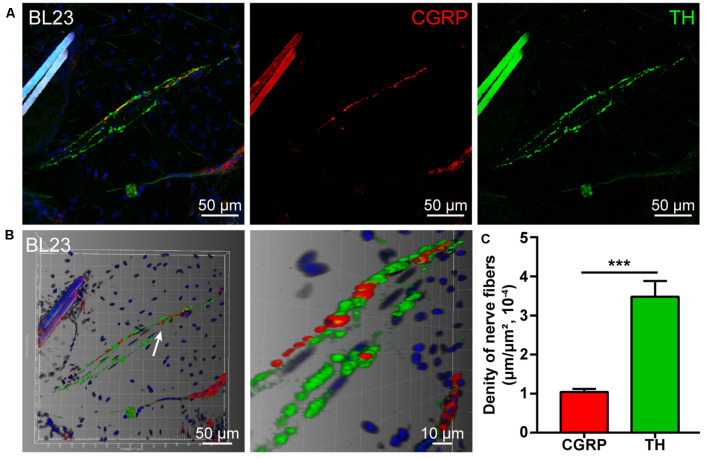
Distribution of the calcitonin gene-related peptide (CGRP)- and tyrosine hydroxylase (TH)-positive nerve fibers in the local tissue of BL23. **(A)** Representative images of CGRP- and TH-positive nerve fibers in the local tissue of BL23. **(B)** Adjusted images from **(A)** with three-dimensional reconstruction in a sloping pattern showing the CGRP- and TH-labeling, respectively. **(C)** The density of CGRP- and TH-positive nerve fibers distributed in the local tissue of BL23 ($\bar{x}$ ± SEM, *n* = 6). ***p < 0.001.

**Figure 2:**
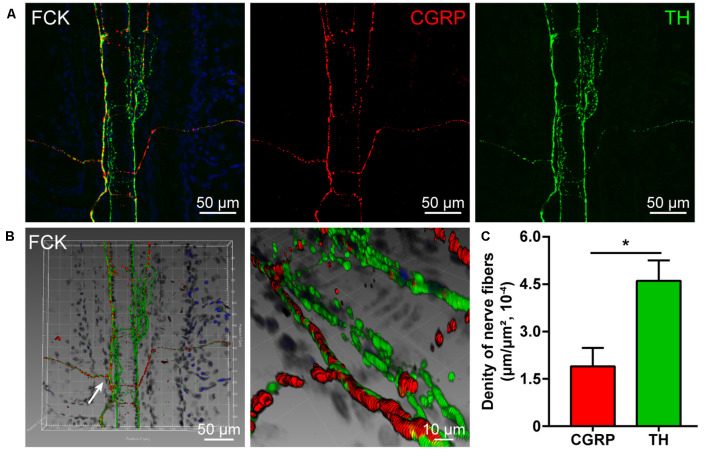
Distribution of the CGRP- and TH-positive nerve fibers in the fibrous capsule of the kidney. **(A)** Representative images of CGRP- and TH-positive nerve fibers in the fibrous capsule of the kidney. **(B)** Adjusted images from panel **(A)** with three-dimensional reconstruction in a sloping pattern showing the CGRP- and TH- labeling, respectively. **(C)** The density of CGRP- and TH-positive nerve fibers distributed in the fibrous capsule of the kidney ($\bar{x}$ ± SEM, *n* = 6). *p < 0.05. FCK refers to the fibrous capsule of the kidney.

By using double fluorescent immunohistochemistry with CGRP and TH, there were abundant CGRP- and TH-positive nerve fibers observed in acupoint BL23 and the fibrous capsule of the kidney (Figures 1A, 2A). These two kinds of nerve fibers ran together or separately. Although some of them are located closely in an intermingling pattern, by three-dimensional analysis, these two types of nerve fibers are distributed independently of each other (Figures 1B, 2B). Furthermore, the length of nerve fibers was quantitatively analyzed within the same twenty areas (2,790 μm × 2,091 μm). As a comparison, the density of TH-positive nerve fibers was significantly higher than that of CGRP-positive nerve fibers in both regions (Figures 1C, 2C).

### The Distribution of the Labeled Neurons With AF488/594-CTB

**Figure 3:**
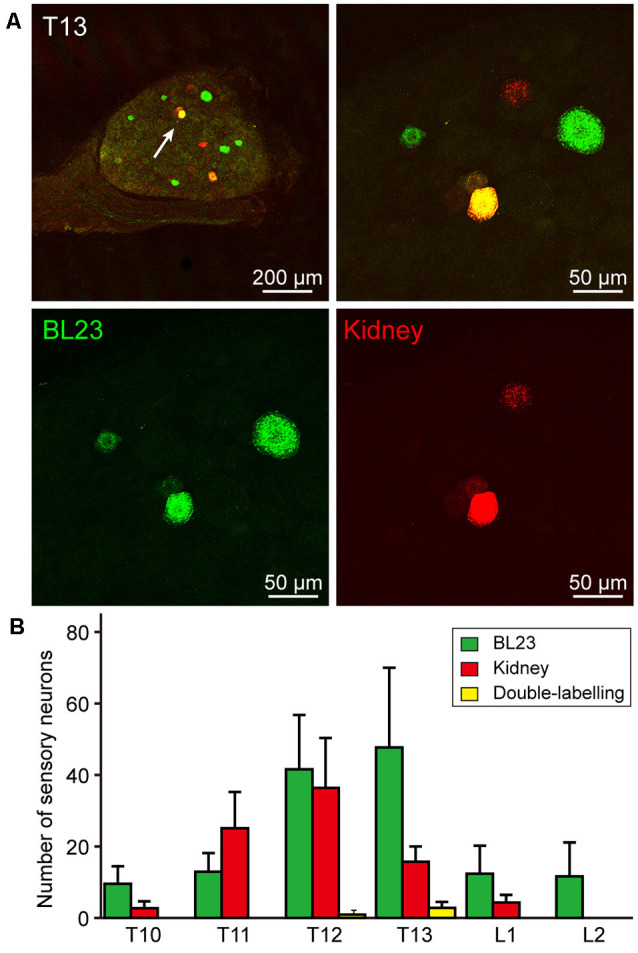
Sensory neurons associated with the acupoint BL23 and kidney in the dorsal root ganglia (DRGs). **(A)** A representative and magnified (arrowhead) photomicrograph showing the distribution of AF488/594-CTB labeled sensory neurons in T13 DRG. The double-labeled neurons with AF488/594-CTB presenting in yellow. **(B)** The number of labeled sensory neurons in the thoracic (T) and lumbar (L) DRGs ($\bar{x}$ ± SEM, *n* = 8).

The sensory neurons associated with acupoint BL23 were labeled with AF488-CTB and observed from thoracic (T) 10 to lumbar (L) 2 DRGs with a higher concentration in T12-T13 DRGs. While the sensory neurons related to the kidney were labeled with AF594-CTB and detected from T10 to L1 DRGs with a higher concentration in T13 DRG. Comparatively, some sensory neurons were simultaneously labeled with AF488/594-CTB in T12-T13 DRGs (Figures 3A,B).

**Figure 4:**
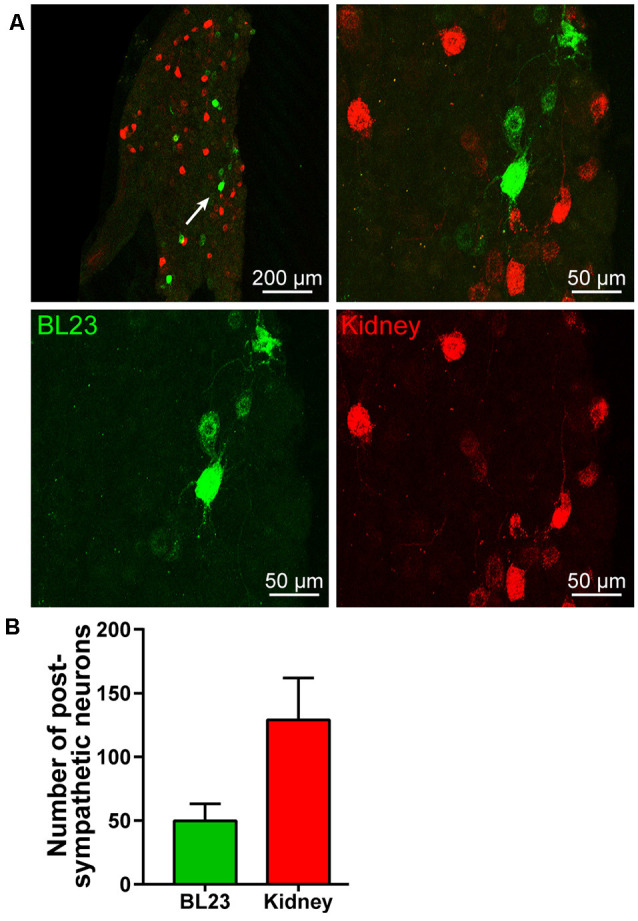
Postympathetic neurons associated with the acupoint BL23 and kidney in the sympathetic chain. **(A)** A representative and magnified (arrowhead) photomicrograph showing the distribution of AF488/594-CTB labeled post sympathetic neurons in the sympathetic chain at the lumbar segments. **(B)** The number of labeled post sympathetic neurons in the sympathetic chain ($\bar{x}$ ± SEM, *n* = 8).

Also, postganglionic neurons associated with acupoint BL23 and kidney were found in the sympathetic chain at the same lumbar segments, but they were separately labeled with AF488-CTB or AF594-CTB (Figure 4A). By counting the labeled neurons, the number of the postganglionic neurons associated with the kidney was higher than that of BL23 (Figure 4B).

**Figure 5:**
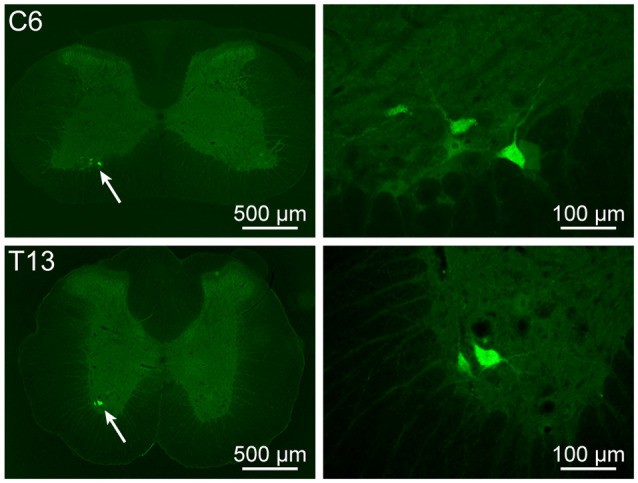
Motor neurons associated with the acupoint BL23 in the spinal ventral horn. The representative and magnified (arrowhead) photomicrographs showing the distribution of AF488-CTB labeled motor neurons at cervical (C) 6 and thoracic (T) 13 segments.

Besides the sensory and sympathetic innervation, the motor innervation was also examined. The motor neurons associated with the acupoint BL23 were also observed in the spinal ventral horn at cervical, thoracic, and lumbar segments, respectively (Figure 5). The number of labeled motor neurons was not further counted in the present study. As a technical limitation, parasympathetic innervation was not demonstrated in the present study.

## Discussion

By applying the immunohistochemical and neural tracing techniques, we provide detailed information to insight into the sensory and sympathetic innervation associated with acupoint BL23 and kidney, including the nerve fibers in local tissues, sensory neurons in the DRGs, and sympathetic neurons in the paravertebral chain.

### Technical Consideration

From a technical point of view, the double fluorescent immunohistochemistry and neural tracing techniques have been effectively used in the field of acupuncture research (Wu et al., 2015b; Wang et al., 2018a, 2019a). Taking the advantages of double fluorescent immunohistochemistry, it makes a sharp contrast between the CGRP- and TH-positive nerve fibers on the local tissue of acupoint BL23 and the fibrous capsule of the kidney. Since the innervations of both the kidney and its fibrous capsule come from the same origin by way of the renal plexus (Mulder et al., 2013; Mompeo et al., 2016; van Amsterdam et al., 2016), the nerve fibers in the kidney were not further examined in the present study. A previous study has shown that CGRP- and TH-positive nerve fibers were distributed widely in the renal parenchymal (Mulder et al., 2013).

For the neural tracing approach, AF488/594-CTB have been frequently used to compare the sensory and motor innervations of the different acupoints as well as the sensory and sympathetic innervations of visceral organs (Cui et al., 2013; Wu et al., 2015a; Zhang et al., 2015, 2018). Using the same approach, we further revealed the innervated characteristics of acupoint BL23 and kidney individually, and also outlined the neuronal correlation between them *via* the sensory and sympathetic pathways.

It should be noted here that the labeled neurons with AF594/488-CTB belong to the first-order neurons associated with the acupoint BL23 and kidney. Although numerous studies have shown that the neurons in the nucleus tractus solitarius can be activated by acupuncture stimulation (Fang et al., 2017; Xiao et al., 2018), these higher-order neurons cannot be traced in this study, which might be transsynaptically traced with neurotropic viruses (Wyss and Donovan, 1984; Weiss and Chowdhury, 1998; Holt et al., 2019). The other limitation of this study should be also emphasized here that, since non-acupoints are still a controversial issue, a control group of non-acupoints was not included in our study (Kim et al., 2017; Liu, 2019).

### CGRP- and TH-Positive Nerve Fibers Associated With the Acupoint BL23 and Kidney

By this study, it is clearly shown that the chemical innervation of the local tissue of acupoint BL23 and the fibrous capsule of the kidney includes the CGRP- and TH-positive nerve fibers. As a comparison, CGRP and TH are expressed separately on different kinds of nerve fibers running in a parallel or individual pattern.

As a sensitive bio-marker, CGRP expresses on the sensory nerve fibers that are widely distributed in the skin and visceral tissues (Eftekhari et al., 2013; Carr and Frings, 2019; Li et al., 2019; Smolilo et al., 2020). Previous studies have shown that CGRP-positive nerve fibers originate from the sensory neurons in DRGs and distribute widely in the local skin tissues of acupoints and the visceral tissues (Ha et al., 2014; Fan et al., 2018; De Logu et al., 2019). Growing evidence indicates that upregulated CGRP can cause vasodilatation and neurogenic inflammation during the pain and plays a critical role in the development of peripheral and central sensitization through the nociceptive signaling pathway (Russell et al., 2014; Iyengar et al., 2017). For the TH, it is the rate-limiting enzyme in the biosynthesis of the catecholamines dopamine, noradrenaline (NA, also known as norepinephrine), and adrenaline (Briggs et al., 2013; Dickson and Briggs, 2013). In the sympathetic nervous system, NA stores in the postganglionic neurons and their nerve fibers with TH-positive expression, serving as the main neurotransmitter released from the sympathetic nerve endings for controlling vasoconstriction (Mukouyama, 2014; Connolly et al., 2015). Accordingly, TH-positive nerve fibers distribute extensively in cutaneous tissues and internal organs (Rodionova et al., 2016; Enríquez-Pérez et al., 2017; Zhang et al., 2020).

Taken together, it indicates that NA and CGRP are a pair of opposite messengers for regulating the vasoconstriction and vasodilatation in response to the local stimulation of the peripheral sensory and sympathetic nerve fibers in the skin (Hodges and Johnson, 2009; Thomas, 2011; Zhang et al., 2020). Although both NA and CGRP play significant roles in maintaining the balance of homeostasis, how the stimulation of the acupoint to affect the release of these chemical messengers in its corresponding visceral organ remains to be studied under the physiological and pathological conditions.

### Neuronal Correlation Between the Acupoint BL23 and Kidney

**Figure 6:**
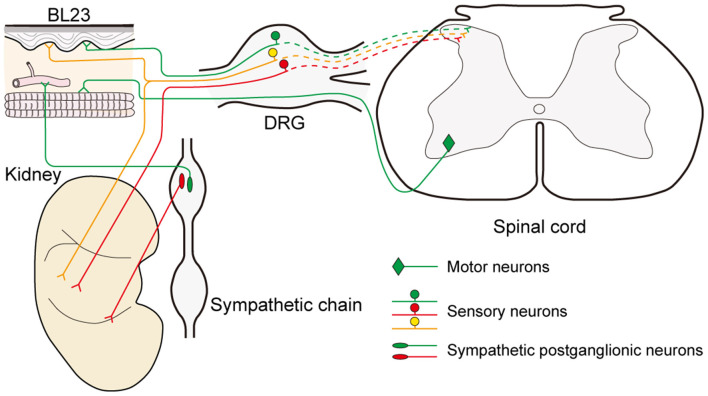
Illustration of the sensory, sympathetic, and motor correlation between the acupoint BL23 and kidney.

In parallel to observing the distribution of the sensory and sympathetic nerve fibers, the origins of sensory and postganglionic neurons associated with the acupoint BL23 and kidney were further determined with the neural tracing approach in this study. Even though most of the neurons were separately labeled with AF488-CTB or AF594-CTB, they locate adjacently in the DRGs and sympathetic chain at the same spinal segments, and some of the sensory neurons were simultaneously labeled with both AF488/594-CTB (Figure 6). These results indicate that there are a direct sensory correlation and an indirect sympathetic relationship between the acupoint BL23 and the kidney. Considering the sensory and sympathetic correlation, it is reasonable to speculate that stimulating signals from the acupoint BL23 area could be either directly or indirectly transported along the sensory and sympathetic pathways to the kidney, conversely, the disorders of the kidney might also be reflected on the body surface through the same routines. A similar neuronal correlation was also observed between the acupoint BL23 and the adrenal gland in our previous study (Zhang et al., 2018). In the present research, we provide another example to explain the neuronal correlation between the Back-Shu points and their corresponding visceral organs *via* the sensory and sympathetic pathways.

Besides the sensory and sympathetic innervations, we also observed the motor neurons that are relative to acupoint BL23 only, and not to the kidney. Additionally, other parasympathetic elements, such as the vagal dorsal motor nucleus and nodose ganglion were not examined in the present study, but it is an important issue to be solved in the future study.

### Clinical Implication

In general, the selection of acupoints along the course of meridians is the basic principle in the treatment of acupuncture. Unlike acupoints on the extremities to be orderly arranged in twelve meridians, it is an exception for the application of Back-Shu Points, because all of them belong to the bladder meridian of Foot-Taiyang, but each of them is responsible for reflecting and treating the disorder of visceral organs in corresponding to its name (Jia and Zhao, 2005). As it is known, certain areas of the skin sensitively responding to visceral diseases has been recorded in ancient Chinese medical texts (Cui X. et al., 2019), while the similar phenomenon has also been systematically investigated by the English neurologist Head (1861–1940) basing on the clinical findings of visceral disease associated with cutaneous manifestations as hyperalgesia or allodynia, and termed these areas as “Head zones” (Head, 1893). A recent study indicated that Back-Shu Points coincide spatially and functionally with the “maximum points” as described within the Head zones (Beissner et al., 2011; Beltrán Molano et al., 2014). According to the anatomo-functional correlation between the Back-Shu Points or the maximum points with their corresponding visceral organs, the most often considered explanation for their interactions is the viscerocutaneous reflexes (Gao et al., 2010; Rong et al., 2011). Although the exact mechanism behind viscerocutaneous reflexes is far from being fully understood, our neuroanatomical evidence supports the idea that viscerocutaneous reflexes could be an important approach for the neuronal interactions between the acupoint BL23 and kidney, which might be started as early as at the level of DRGs and sympathetic chain.

Coincidently, the “stimulating peripheral activity to relieve conditions (SPARC)” program to be funded by the National Institutes of Health is also aimed to investigate the complex pathways of nerve-organ interactions for ultimately promoting the precise treatment of diseases and conditions through peripheral electro-stimulation. Both acupuncture and SPARC are established by way of stimulating the peripheral nervous system. Therefore, figuring out the correlation between two types of peripheral nerves, somatic and visceral, could serve as a great source of inspiration for modern acupuncture research. As representative targets of acupoint and visceral organs, our study in the neuronal correlation of BL23 and kidney may provide an example to examine the interconnection between somatic and visceral nerves.

Increasing evidence shows that visceral diseases can induce the referred pain or sensitization on the body surface at their corresponding spinal segments in patients and experimental rats, which were closely correlated with the locations of traditional acupoints (Chen et al., 2014; Shi et al., 2018). In facing the sophisticated neuronal interconnection between the acupoints and visceral organs, from the viewpoint of the neural pathway, Back-Shu Points could be an important starting for observing the neuronal correlation with their corresponding visceral organs. The precise neural network how to serve the signal communication between the Back-Shu Points and their corresponding visceral organs might be a new issue in future studies.

In summary, by using the double fluorescent immunohistochemistry and neural tracing techniques, we have provided histological evidence to benefit the understanding of the sensory and sympathetic innervation of the acupoint BL23 and kidney. Considering the chemical characteristics and neural pathways, the acupoint BL23 and kidney establish a close sensory and sympathetic correlation between each other. Although present data cannot explain how the acupoint BL23 works on the kidney, this neuroanatomical connectivity may provide clues for exploring the mechanism of the role of neural pathways between the acupoint BL23 and kidney, which might also provide insights into the general rules underlying the other Back-Shu Points and their corresponding visceral organs.

## Data Availability Statement

The original contributions presented in the study are included in the article, further inquiries can be directed to the corresponding author/s.

## Ethics Statement

The animal study was reviewed and approved by ethics committee of the Institute of Acupuncture and Moxibustion, China Academy of Chinese Medical Sciences.

## Author Contributions

The manuscript presented here was carried out in collaboration between all authors. ZZ, DX, SW, YS, and LZ carried out the experiments. ZZ, DX, JW, and JC analyzed the data. XJ and WB designed the study. ZZ, JW, and WB wrote the article. All authors read and approved the final version of the manuscript accepted for publication.

## Conflict of Interest

The authors declare that the research was conducted in the absence of any commercial or financial relationships that could be construed as a potential conflict of interest.
